# Effects of Subjective Memory Complaints (SMCs) and Social Capital on Self-Rated Health (SRH) in a Semirural Malaysian Population

**DOI:** 10.1155/2019/9151802

**Published:** 2019-04-10

**Authors:** Kwong Hsia Yap, Devi Mohan, Blossom C. M. Stephan, Narelle Warren, Pascale Allotey, Daniel D. Reidpath

**Affiliations:** ^1^Jeffrey Cheah School of Medicine and Health Sciences, Monash University Malaysia, Jalan Lagoon Selatan, Bandar Sunway, Subang Jaya, 47500 Selangor, Malaysia; ^2^Newcastle University Institute for Ageing and Institute for Health & Society, Newcastle University, Newcastle Upon Tyne NE4 5PL, UK; ^3^School of Social Sciences, Clayton Campus, Monash University, Clayton VIC 3800, Australia; ^4^International Institute for Global Health, United Nations University, Kuala Lumpur, Malaysia; ^5^South East Asia Community Observatory (SEACO), Monash University, Segamat, Malaysia

## Abstract

Subjective memory complaints (SMCs) and social capital were known to be related to self-rated health (SRH). Despite this, no studies have examined the potential interaction of SMC and social capital on SRH. Using data from a cross-sectional health survey of men and women aged 56 years and above (*n* = 6,421), we examined how SMCs and social capital explained SRH in a population of community-dwelling older adults in a semirural area in Malaysia. We also evaluated whether SRH's relationship with SMCs is moderated by social capital. The association of SMC and social capital with poor SRH was investigated using multivariable logistic regression. Social capital (OR = 0.86, 95% CI = 0.82–0.89), mild SMC (OR = 1.70, 95% CI = 1.50–1.94), and moderate SMC (OR = 1.90, 95% CI = 1.63–2.20) were found to be associated with poor SRH after adjustment for sociodemographic factors and depression in the initial regression model. SMC was found to have partial interaction effects with social capital which was included in the subsequent regression model. Unlike individuals with no SMC and mild SMC, those who reported moderate SMC did not show decreasing probabilities of poor SRH despite increasing levels of social capital. Nevertheless, this analysis suggests that social capital and SMC are independent predictors of poor SRH. Further research needs to be targeted at improving the understanding on how social capital and SMC moderate and interact with the perception of health in older adults.

## 1. Introduction and Background

Subjective memory complaints (SMCs) are self-reported problems with memory that may or may not present with objective cognitive impairment (measured via tests and assessments). SMC is common in the elderly, and its prevalence can be as high as up to 50% in older adults [1–3]. The aetiology of SMC can be heterogeneous in nature ranging from poor function in daily activities, low mood, personality traits, and even neurodegenerative disorders [4–6]. Of particular interest is SMC's link to dementia. SMC has been proposed as the first subtle sign of decline before the appearance of preclinical dementia and even before actual objective cognitive impairment [7, 8]. People with SMC showed evidence of neuropathological changes associated with Alzheimer's disease (AD) as well as with low levels of amyloid-*β* in cerebrospinal fluid, which predicted the progression of AD [9]. In some individuals, SMC may reflect mood (e.g., depression) rather than cognitive symptoms [10–12]; however, memory complaint appears to have continued predictive value for cognitive decline even after controlling for depression [13]. While not every individual with SMC will develop AD or dementia, the risk of dementia in individuals with SMC (especially in individuals without objective impairment) is higher [14]. There is also a growing interest in the role of subjective memory complaints (SMCs) as a predictor of future diseases in older people including cognitive impairment [15] and stroke [16] because it is quick and simple to measure. A life course approach linked how shared risk factors in early life may have led to cognitive dysfunction and chronic diseases [17], where the latter may also contribute to the risk of dementia.

SMC is also associated with poor self-rated health (SRH) [18–20]. SRH is a subjective assessment of one's health which is known as a useful indicator of health status [21]. In the elderly, SRH is known to predict a number of health outcomes including mortality [22], cognitive impairment [23], dementia [24, 25], and even functional decline [26]. The simplicity and predictive properties of SRH are the main reasons it is widely used as an overall measure of general health [21]. The presence of SMC in the elderly was predicted and was associated with the increase in the utilization of health services [6, 27]. The increase in health service utilization was mainly due to reduced function resulting from the concurrent presentation of chronic diseases (alongside SMC) [6] and increased nursing home placement [27]. The decrease in functionality was suggested to result in poorer physical health, and this may be what affected a person's SRH.

Social capital is known for its positive relationship with SRH. Studies have consistently found that higher levels of social capital are associated with good SRH and vice versa [28, 29]. Social capital is a multidimensional concept that can be broadly defined as the social resources from the individual or group social relationships that can be gained and used to reach individual or collective goals [30–32]. The main features of the social relationships that provide such resources are trust and norms of reciprocity. Those with higher levels of social capital are generally found to be more involved in their communities, socially engaged with friends and neighbours, and have a higher likelihood of trusting and thinking kindly of others [33]. Social engagement and active community involvement (dimensions of social capital) have also been associated with increased life expectancy and better physical and mental health [34, 35]. Social capital is also postulated to be protective of cognitive function as social networks and activities encourage social interaction and exchange which in turn provides stimulation and mediates depression [36–38].

Low- and middle-income countries (LMICs) are experiencing population ageing [39] and facing the burden of increase in chronic diseases [40] as well. It is estimated that the greatest burden of dementia now lies in LMICs [41]. Social capital is an important determinant of health, especially in low human and financial capital settings (such as in LMICs), but there is a lack of literature on social capital from LMICs [42, 43]. Therefore, there is a need to expand the knowledge base beyond high-income countries and select middle-income countries as social capital's well-known relationship with SRH may well serve as a resource for the elderly in LMICs. Similar to social capital, a key limitation of previous population-based studies on SMC is that they have focused mainly on older populations in high-income and Western countries [2, 44–47]. Studies from Asia came from high-income countries in Asia [48–50]. Given SMC's association with poor SRH and other health outcomes, the lack of research in this area is problematic as SMC is common in older people. This also raises an important question if social capital is able to attenuate the effects of SMC on SRH.

In this study, we aim to examine the effects of social capital and SMC on SRH and to investigate if social capital has attenuating effects on SMC's relationship with SRH. The study was conducted in a semirural community setting among older aged adults in Malaysia.

## 2. Methods

The study relies on a secondary analysis of cross-sectional data extracted from a database of older aged individuals who had previously completed a household survey including a health assessment.

### 2.1. Participants

Participants were part of a larger cohort study in the South East Asia Community Observatory (SEACO), a health and demographic research surveillance site situated in Malaysia. Malaysia is a multiethnic middle-income country undergoing rapid population ageing and demographic transitions, but these developments were not balanced by adequate health and financial policies to reflect the needs resulting from these transitions [51]. Full methodological details of SEACO have been published previously [52, 53]. The SEACO surveillance area is largely semirural and comprises urban, rural, and plantation areas with an ethnic mix of Malays, Chinese, and Indians, close to national proportions of Malaysia [54]. 8,496 individuals aged 56 years and older were approached to complete a cross-sectional health survey including sociodemographic information (e.g., age, gender, education, finances, and domicile), health-related conditions (e.g., illnesses, depression, anxiety, and stress), lifestyle habits (e.g., smoking, alcohol, physical activity, and social connectivity), and quality of life. The survey was conducted between August 2013 and March 2014 and achieved an 80.3% response rate. All interviews were conducted at the participants' usual place of residence by local community-based data collectors. All participants provided written consent prior to data collection.

### 2.2. Measures

SMC was measured using the memory item from the WHO Study on global AGEing and adult health (SAGE) [55] on a 5-point Likert scale (none, mild, moderate, severe, and extreme.) The item was “Overall in the last 30 days, how much difficulty did you have with concentrating or remembering things?” Less than 2% of the participants responded as “severe” and “extreme.” In light of that, these responses were combined into the group who responded “moderate.” The final SMC variable used in this analysis consisted of three levels; “no SMC,” “mild SMC,” and “moderate SMC.”

SRH was measured using the five-point rating scale from the WHOQOL-BREF [56] item: “How satisfied are you with your health?” The item was dichotomised into “poor” (very dissatisfied, dissatisfied, and neither satisfied nor dissatisfied) and “good” SRH (satisfied and very satisfied). This cutoff was chosen based on the heavily skewed distribution of the responses where more than 60% of the participants indicated that they were satisfied and very satisfied with their health.

In the absence of agreed, standardised measures of social capital, we have chosen to operationalise it in a way analogous to Kawachi et al. [28], who identified key survey questions representing trust and reciprocal norms in the community. We focused on variables representing trust and reciprocal norms in this survey: attachment “I feel a strong attachment to my local community;” cooperative norms “If I see people who cooperate with each other, I also feel that I would help someone in need;” reciprocity “If I do nice things for someone, I can anticipate that they will respect me and treat me just as well as I treat them;” and community support “In a difficult situation, I can count on the help from people in my local community.” These four items used a 5-point Likert-type scale, coded from lower social capital to higher (1 = totally disagree, 2 = totally disagree, 3 = neither agree nor disagree, 4 = agree, and 5 = totally agree). To reduce the four measures of social capital, we conducted a principal component analysis to derive a social capital index. Preliminary analysis yielded one principle component with eigenvalue greater than one (eigenvalue = 2.8), and it accounted for 70% of total cumulative variance. This principle component was then used in all subsequent regressions.

Depression is known to be correlated with SMC [10, 11] and associated with poor SRH [57] in the elderly. We adjusted for depressive symptoms to tease out the effects of SMC, in the subsequent multivariate analyses. Depression was measured using the Depression, Anxiety and Stress Scale-21 (DASS-21) [58]. Individuals were classified as depressed with the scale developers cutoff score ≥10 [59] used to separate people with normal scores from those with mild depression or worse.

Additional sociodemographic covariates included gender (male (reference group) and female), age (56–64 (reference group), 65–74, and 75 + years), ethnicity (Malay (reference group), Chinese, and Indian), marital status (not married (reference group) and married), education level (no formal education (reference group), 6 years or less, above 6 years, and others (nonspecified)), and employment status (working (reference group), not working/unemployed, and retired/pension).

### 2.3. Statistical Analysis

The proportions of SRH status in each variable were reported as percent. Differences in the participant characteristics according to SRH status were tested using the chi-squared statistic. Binary logistic regression analyses were used to determine the associations between SMC and social capital with poor SRH. Two models were developed: Model 1 tested the association between SMC and social capital with SRH controlling for the sociodemographic covariates, age, ethnicity, gender, education, employment status, marital status, and depression. Model 2 added the measure of social capital and the interaction term of SMC and social capital. Results are reported as odds ratios (ORs) with 95% confidence intervals (95% CIs). The marginal analysis was used to assess and graphed to depict the effect of the interaction term (social capital × SMC) on the outcome of interest (poor SRH) while simultaneously adjusting for all other covariates in the model. All analyses were undertaken using STATA version 14.0 (StataCorp. 2015. Stata Statistical Software: Release 14. College Station, TX: StataCorp LP.).

## 3. Results

The population sample, aged 56 years or older, comprised 6,829 participants. We excluded individuals with incomplete data for any of the variables, resulting in a final sample of 6,421 older adults. That is, only 6% of the total data set contained missing data. An analysis by gender identified no significant differences between those with and without missing data. A higher proportion of participants in the older age group of 75 years and above was also found to have more missing data. The possible implications of this are discussed later. The final analytical sample comprised 3,408 women (53.1%) and 3,013 men (46.9%).

Table 1 shows the characteristics of the study population by SRH status. The distribution of all sociodemographic variables (with the exception of gender) and depressive symptoms showed differences in proportions in the SRH groupings. In total, about two-thirds of the sample reported some levels of memory complaint (39.3% mild SMC; *n* = 2,177 and 22.8% moderate to extreme SMC; *n* = 1,461). Majority of the study population (more than 60%) reported more favourable values (agree and totally agree) in all the social capital variables. All the independent variables of interest (SMC and social capital) showed differences in proportions in the SRH groupings as well.

Table 2 shows the results of the logistic regression modelling. After controlling for the sociodemographic covariates and depression (Model 1), SMC was a significant predictor of poor SRH. Mild SMC (OR = 1.70; 95% CI = 1.50–1.94) and moderate SMC (OR: 1.90; 95% CI = 1.64–2.20) were at higher odds of poor SRH compared to the no-SMC group. In the final adjusted model (Model 2), SMC, social capital, and the interaction of SMC with social capital were found to be significant. The more complex Model 2 had a significantly better fit to the data than Model 1 (Wald test = 30.57, *p* < 0.001).

**Figure 1:**
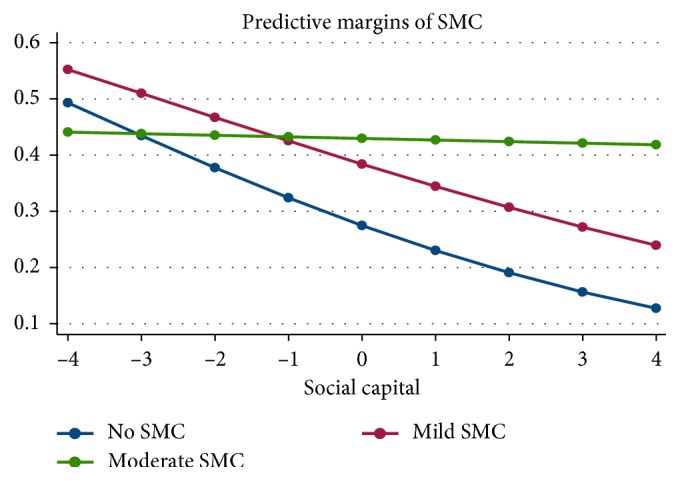
Marginal analysis of the interaction between social capital and SMC on the predicted probability of poor SRH.

Figure 1 illustrates the marginal analysis of the interaction between social capital and SMC on the predicted probability of poor SRH. In the no-SMC and mild SMC groups, increasing levels of social capital resulted in decreasing probability of poor SRH. In the group with moderate SMC, the probability of poor SRH did not exhibit much change regardless of the social capital levels.

## 4. Discussion

The results of the analysis show associations between SMC, social capital, and SRH in an older, semirural population from Malaysia. The greater an individual's social capital, the lower the odds of poor SRH. However, there are differences in the effects of social capital on the SMC groups. In the individuals with no SMC and mild SMC, while increasing social capital is associated with lower probability of poor SRH, the predicted probability of poor SRH remained higher in mild SMC in comparison with individuals who reported no SMC. These results are broadly consistent with earlier studies on the relationships of SMC with poor SRH [19, 60], and that low levels of social capital are associated with poor SRH [28, 29]. This study extended the understanding of the relationships, whereas previous work has examined relationship between SRH and SMC or SRH and social capital separately; the inclusion of social capital and interaction effects that differs with the different levels of SMC underscores the potential complexity of the relationships and the considerations that contribute to SRH.

Interaction effects of social capital was only partial, only for the moderate SMC group, suggesting that the SMC levels of mild and moderate were distinct from one another (Figure 1). With higher levels of social capital, the rate of decrease in moderate SMC was almost negligible compared to no SMC and mild SMC. One reason could be that a higher proportion of people who were identified with having moderate to extremely severe memory complaints had a greater degree of cognitive impairment. People with more severe cognitive impairment may experience higher degrees of anosognosia which affect their self-awareness and capacity to understand their condition [61]. It was found that, in cognitively intact and moderately cognitively impaired older persons, poor SRH was still able to predict mortality but not for those who were severely cognitively impaired [62]. Poor SRH in cognitively impaired older adults did not predict dementia [25]. This suggests that the argument of cognitive processes that influenced self-rated health [21] may be the reason for this. People with more severe cognitive impairment may not be able to integrate less immediate factors that typically contribute to an overall assessment of health [25, 62]. Cognitive deficits too may have also impaired the perception of social capital and have the potential to moderate the effects of social capital on SRH [63]. Another reason could be the interaction of different dimensions of social capital within the study population. The social capital index was derived from variables representing individually perceived norms of trust and reciprocity in the community which represents one dimension of social capital. Certain groups of people with SMC were found to have lowered social interaction [64] and social functioning [65] which may have affected social participation, another dimension of social capital. The interaction of low social participation with high trust in older adults resulted in worse SRH and psychological health which differed by gender [66]. Older adults can retain high social capital but lowered social participation due to a decrease in functionality. However, social participation was not assessed in this study, so this potential interaction was not evaluated. The measurement of SRH may only offer more valuable insight up to SMC moderate reporters.

The strengths of this study include large sample size which allowed the possibility of detecting small effect sizes and population representativeness. The sample size also allowed us to disaggregate the assessment of SMC into multiple levels. Other population studies have primarily assessed SMC (“*Do you have any memory problems*?”) based on a binary response of “yes” or “no” [15] which would not have allowed the detection of the differences between the interactions of social capital with the SMC levels. There are however some weaknesses. First, data on objective cognitive performance was not collected, and therefore it is not possible to determine whether SMC reflects real impairment. However, advantages of using SMC to gain insight into the cognition status of large population studies include low costs and simple and brief administration [67]. In countries with limited resources, detailed objective measurements of cognitive status may not be always possible especially for large population studies. Furthermore, qualitative narratives on dementia [68, 69] and the perception of cognitive health in different cultures [70] have highlighted cultural differences in how people perceive cognitive abilities and states. Moreover, despite the heterogeneity of SMC aetiology, SMC had in many previous studies predicted worse health outcomes in older adults and may be worth monitoring. More data contributions from ethnically diverse countries such as Malaysia is needed to advance the understanding on SMC and its relevance, be it to SRH or other important outcomes such as mortality and dementia. A small proportion of the records (6%) were excluded because of missing data, and it was more likely that older people had missing data—which was consistent with in general for cognitive decline. While it is not expected that the missing data would have a major effect on the broad trends in the data, it is a possibility and this opens avenues for future research.

The identified complexity and the measurement challenges perhaps suggest that what is truly missing here would be the contextual understanding of what is valued by older people in the different settings that they are in and how it contributes to how they perceive their individual health and cognitive states. While ethnicity was used as a control variable in the analysis and was not the core focus of this paper, it is worth noting that there were ethnic differences in terms of SMC proportions (data not shown). These kinds of differences have been observed in other research [71]; however, it is an underexplored area. Qualitative-based enquiries would facilitate these understandings and would offer a more robust framework for the formation of quantitative data collection or surveys later on. There is a significant caveat to this analysis, and it is a caveat that necessarily exists with many studies identifying higher-order interaction effects in health. Because health is a multiattribute domain, many identifiable factors are correlated, such as the various sociodemographic variables, depression, and cognitive performance. The specific approach to the modelling may identify one set of interactions or another. In a cross-sectional analysis, sorting out the causal order cannot be done definitively although one can speculate rationally about it. The problem is not resolved perfectly with longitudinal data because there are often feedback loops that can be masked by a lack of temporal granularity. The choice of health-related factors, how those factors are measured, how they are combined, and how they are followed over time will all influence the interpretation. Notwithstanding these issues, stepping models without interaction effects begins to reveal potentially important lines of inquiry.

Nevertheless, the analysis does suggest that social capital and SMC are independent predictors of poor SRH. In the context of Malaysia and other LMICs experiencing population ageing, this has potentially important implications. SMC is common in older people. Family social support in LMICs has traditionally been expected to provide the main source of caregiving for the elderly [72] in the absence of strong social protection and social welfare. However, changes in the rural demography caused by rural-urban migration has resulted in the rapid “nuclearisation” of families concentrated in urban areas and even more rapid ageing of populations in rural areas [73, 74]. This loss of multigenerational households has reduced the social protection afforded by family. In addition to that, the fragmented healthcare services and lack of support services for chronic diseases in Malaysia [75, 76] (and other LMICs) even in the urban areas leave the rural elderly in a potentially vulnerable state. Within this context, there is a need to understand better how social capital moderates the perception (and reality) of health in older people, even in the more potentially vulnerable SMC moderate reporters.

## 5. Conclusion

SMC and social capital were found to be associated with poor SRH. However, in moderate SMC reporters, levels of social capital did not seem to positively affect the probability of poor SRH. Against the backdrop of the evolving social structure in LMICs, there is a need to better understand how social capital contributes to SRH in the elderly, particularly in the elderly who are SMC reporters.

## Figures and Tables

**Table 1:** Characteristics of study population (*n* = 6421).

Variable	Total *n* (%)	Good SRH *n* (%)	Poor SRH *n* (%)	*p* value
Gender				0.186
Female	3408 (53.1)	2182 (52.4)	1226 (54.3)	
Male	3013 (46.9)	1979 (47.6)	1034 (45.7)	
Ethnicity				<0.001
Malay	4048 (63.0)	2720 (65.4)	1328 (58.8)	
Chinese	1853 (28.9)	1084 (26.0)	769 (34.0)	
Indian	520 (8.10)	357 (8.6)	163 (7.2)	
Age				<0.001
56–64	3493 (54.4)	2364 (56.8)	1129 (50.0)	
65–74	2029 (31.6)	1301 (31.3)	728 (32.2)	
75 above	899 (14.0)	496 (11.9)	403 (17.8)	
Education				<0.001
No formal education	340 (5.3)	187 (4.5)	153 (6.8)	
6 years and below	3778 (58.8)	2420 (58.2)	1358 (60.1)	
Above 6 years	1995 (31.1)	1354 (32.5)	641 (28.3)	
Other	308 (4.8)	200 (4.8)	108 (4.8)	
Employment				<0.001
Working	2104 (32.8)	1481 (35.6)	623 (27.6)	
Unemployed	3439 (53.5)	2129 (51.2)	1310 (57.9)	
Retired/pension	878 (13.7)	551 (13.2)	327 (14.5)	
Marital status				<0.001
Not married	1532 (23.9)	936 (22.5)	596 (26.4)	
Married	4889 (76.1)	3225 (77.5)	1664 (73.6)	
Depressive symptoms				<0.001
No	5362 (83.5)	3697 (88.9)	1665 (73.7)	
Yes	1059 (16.5)	464 (11.1)	595 (26.3)	
SMC				<0.001
No	2434 (37.9)	1828 (43.9)	606 (26.8)	
Mild	2526 (39.3)	1541 (37.0)	985 (43.6)	
Moderate to extreme	1461 (22.8)	792 (19.0)	669 (29.6)	
Reciprocity				<0.001
Totally disagree	37 (0.6)	25 (0.6)	12 (0.5)	
Disagree	497 (7.7)	319 (7.7)	178 (7.9)	
Neither agree nor disagree	1539 (24.0)	733 (17.6)	806 (35.6)	
Agree	3664 (57.1)	2580 (62.0)	1084 (48.0)	
Totally agree	684 (10.6)	504 (12.1)	180 (8.0)	
Cooperative norms				<0.001
Totally disagree	26 (0.4)	20 (0.5)	6 (0.3)	
Disagree	270 (4.2)	156 (3.7)	114 (5.0)	
Neither agree nor disagree	1435 (22.4)	684 (16.4)	751 (33.2)	
Agree	3989 (62.1))	2787 (67.0)	1201 (53.2)	
Totally agree	701 (10.9)	514 (12.4)	187 (8.3)	
Community support				<0.001
Totally disagree	61 (0.9)	30 (0.7)	31 (1.4)	
Disagree	502 (7.8)	283 (6.8)	219 (9.7)	
Neither agree nor disagree	1626 (25.3)	807 (19.4)	819 (36.2)	
Agree	3696 (57.6)	2636 (63.4)	1060 (46.9)	
Totally agree	536 (8.4)	405 (9.7)	131 (5.8)	
Attachment/belonging				<0.001
Totally disagree	71 (1.1)	40 (1.0)	31 (1.4)	
Disagree	381 (5.9)	207 (5.0)	174 (7.7)	
Neither agree nor disagree	1847 (28.8)	924 (22.2)	923 (40.8)	
Agree	3590 (55.9)	2582 (62.0)	1008 (44.6)	
Totally agree	532 (8.3)	408 (9.8)	124 (5.5)	

**Table 2:** Logistic regression analyses assessing the relationship between SMC, social capital, and poor SRH. All models were controlled for gender, ethnicity, age, education, employment, marital status, and depression.

	Odds ratio (95% confidence interval) for poor SRH	
	Model 1	Model 2
SMC		
Mild	1.70 (1.50–1.94)^*∗∗*^	1.81 (1.57–2.09)^*∗∗*^
Moderate	1.90 (1.64–2.20)^*∗∗*^	2.47 (2.09–2.92)^*∗∗*^
Social capital	0.86 (0.82–0.89)^*∗∗*^	0.82 (0.77–0.87)^*∗∗*^
Interactions		
Social capital × SMC		
Mild		1.07 (0.99–1.16)
Moderate		1.26 (1.16–1.37)^*∗∗*^

^*∗∗*^p < 0.001. Model 1: the effect of SMC and social capital on poor SRH controlling for sociodemographic factors and depression; Model 2: Model 1 + interaction term: SMC × social capital.

## Data Availability

The data used to support the findings of this study are available from the corresponding author upon request.
